# Habitat selection and diurnal refugia of gray foxes in southwestern Georgia, USA

**DOI:** 10.1371/journal.pone.0186402

**Published:** 2017-10-17

**Authors:** Nicholas R. Deuel, L. Mike Conner, Karl V. Miller, Michael J. Chamberlain, Michael J. Cherry, Larry V. Tannenbaum

**Affiliations:** 1 Joseph W. Jones Ecological Research Center, 3988 Jones Center Dr., Newton, Georgia, United States of America; 2 Warnell School of Forestry and Natural Resources, University of Georgia, Athens, Georgia, United States of America; 3 Army Public Health Center, MCHB-PH-HRA, Building 1675, Aberdeen Proving Ground, Maryland, United States of America; Smithsonian Conservation Biology Institute, UNITED STATES

## Abstract

Understanding habitat selection of gray foxes (*Urocyon cinereoargenteus*) is essential to evaluate their potential response to changes in land use and predator communities. Few studies have evaluated temporal habitat selection or explicitly identified habitats used by gray foxes for diurnal refugia. We used GPS collars to obtain location data for 34 gray foxes (20 males and 14 females) from February 2014 to December 2015 to evaluate temporal (seasonal and diel) habitat selection and selection of diurnal refugia in southwestern Georgia, USA. We analyzed habitat selection at 2 levels, selection of a core area within the home range and selection of locations within the home range. Habitat selection was non-random (*P* < 0.001) but consistent among seasons, between day and night, and between sexes (*P* > 0.05). Hardwoods, human use (i.e., areas associated with regular human activity such as buildings, lawns, parking areas, etc.), and roads were selected (*P* < 0.05), whereas pine dominated stands were used randomly (*P* > 0.05). Selection of habitats for diurnal refugia did not vary seasonally or by sex (*P* > 0.05), with foxes selecting (*P* < 0.05) areas near hardwood forests, roads, agriculture, human use, pastures/food plots, and shrub scrub habitats. Gray foxes were observed on the ground while resting, and we found no evidence of gray foxes diurnally resting in trees. Our results suggest that on our study area, gray foxes are an edge species that prefer forests with a hardwood component in areas near human use and roads.

## Introduction

Over the last two centuries, North America has experienced a rearrangement of predator communities largely induced by extirpation of gray wolves (*Canis Lupus*; [1], [2]). As a result, coyotes (*Canis latrans*) have expanded their distribution, abundance, and ecological influence on North American food webs. Increases in abundance of coyotes are often associated with the suppression of foxes (*Vulpes vulpes*, *V*. *velox*, *Urocyon cinereoargentus* [1], [2]). Suppression of foxes by coyotes is often heralded as a conservation achievement that can result in increased mammalian [3] and avian biodiversity [4], and waterfowl nest success [5]. However, there are species of foxes that are of conservation concern (e.g., San Joaquin kit fox; *Vulpes macrotis mutica*) [6] and this pressure from expanded coyote populations often creates challenges for management of these species. A more complete understanding of the spatial ecology of foxes persisting in coyote-dominated landscapes may illuminate habitat associations important to conservation of native fox species that historically were likely more abundant before the collapse of wolves and the expansion of coyotes [1], [2].

Gray foxes (*Urocyon cinereoargenteus*), as with all species, must balance behavioral choices to obtain food and successfully reproduce while avoiding predation. Therefore, gray foxes should select habitats that increase fitness [7] by balancing predation risk and foraging opportunities [8]. Thus, land use conditions with abundant and available prey [9] and ample escape cover that reduces probability of encounter with predators [10], [11] are likely preferred. However, desirable habitat attributes may not be present in a single land use type [12]. Most importantly, food and cover requirements vary temporally, but few studies have evaluated habitat selection relative to time of day or activity status (i.e., active or inactive) [13]. Although gray fox habitat selection has been studied in many portions of their geographic range [9], [10], [14], recent technological advances (i.e., smaller batteries and Global Positioning System (GPS) receivers) allow locations with greater precision and accuracy [15], and because automated sampling occurs at programmed intervals, it is now possible to collect sufficient data to robustly compare habitat selection between day and night, and more easily identify and evaluate refugia.

Prior studies have reported that gray foxes select hardwood forests [6], [10], [16], mixed pine-hardwood forests [9], [17], mature pine forests [9], mid-rotation pine plantations [14], and areas close to human dwellings [10], [11], [18]. Differences in habitat selection across studies likely reflect site-specific differences in habitat availability, prey abundance [9], forage availability [19], and predator communities [20], [21]. Gray fox habitat selection also varies seasonally with availability of resources. For example, gray foxes consume soft mast, small mammals, birds, and insects [22], all of which vary seasonally in availability [22], [23]. Some studies suggest seasonal differences in gray fox habitat selection [14], [24], whereas others do not [9], [10].

Fine-scale temporal (e.g., nocturnal versus diurnal) assessment of habitat selection has not been explicitly evaluated in gray foxes. Instead, prior studies provided anecdotal descriptions of diurnal and nocturnal habitat selection without explicitly quantifying if selection differs between the 2 periods. These studies suggest gray foxes are more often found in habitat types with dense brushy understories during the day [14], [25], [26] whereas at night habitat selection does not occur [14], [25] or it occurs within open habitat types [18], [24], [26]. Coyotes and bobcats (*Lynx rufus*) are known to prey on gray foxes [10], [27] and are generally ubiquitous within the geographical range of the gray fox. Differences between nocturnal and diurnal habitat selection by gray foxes likely reflect balancing predation risk with foraging opportunities [28]; thus, enhanced understanding of finer-scale temporal habitat preferences should provide greater insight on habitat preferences of gray foxes. Therefore, we used GPS technology to evaluate temporal influences (i.e., diurnal vs. nocturnal, and seasonal) on gray fox habitat selection at 2 spatial scales in a coyote-dominated landscape in southwestern Georgia. We also used this technology to infer location of gray fox diurnal refugia and quantified habitat associated with these sites.

## Methods

### Study area

**Fig 1:**
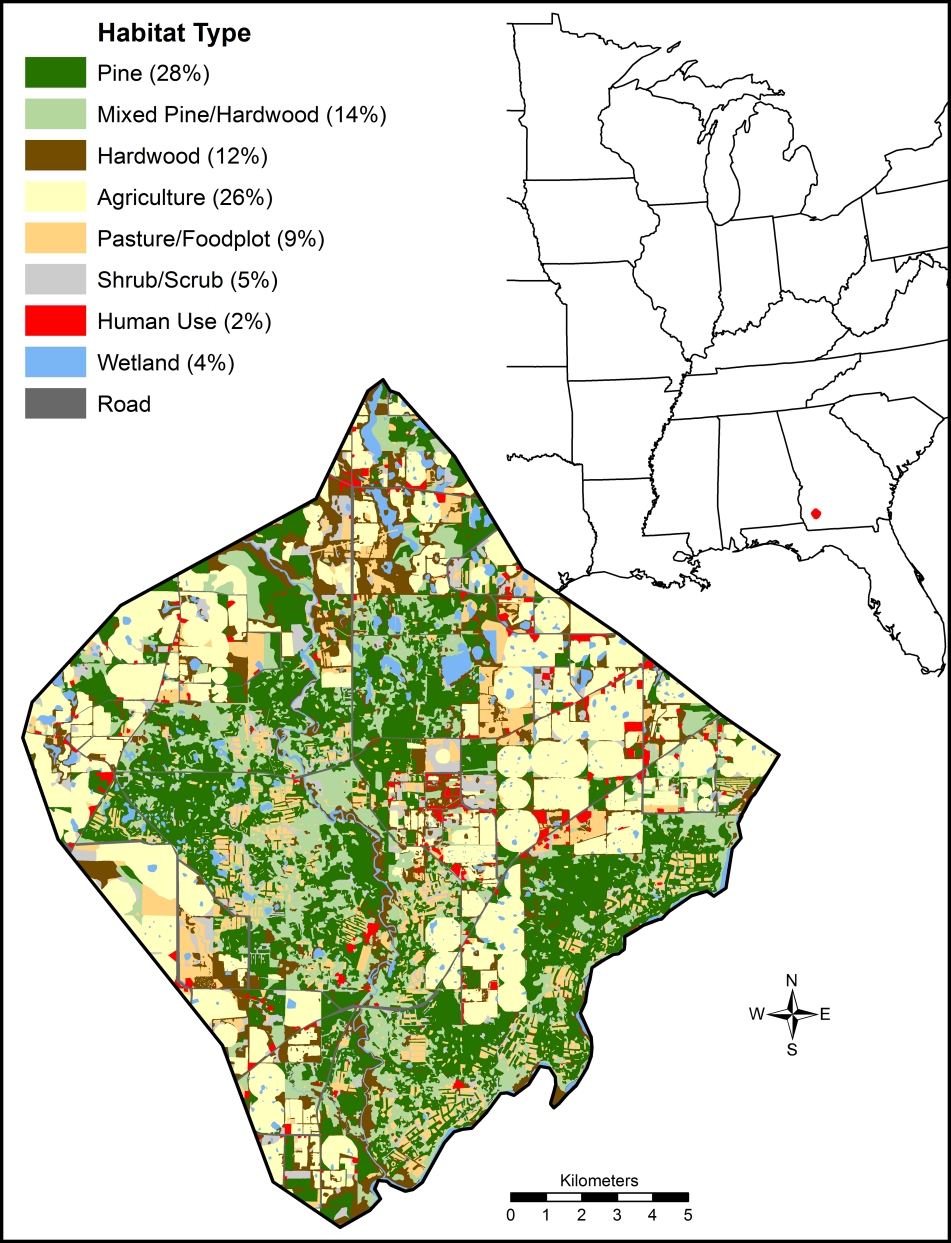
Location, spatial arrangement, and percentage of vegetation types in Southwestern Georgia, USA at a site used to study gray fox habitat selection from February 2014 to December 2015.

We conducted our study at the Joseph W. Jones Ecological Research Center at Ichauway, and surrounding lands in Baker County, Georgia, USA (Fig 1). Topography was mostly flat, with elevation ranging from 27–200 m above sea level. Climate was subtropical with hot, humid summers and mild, wet, and short winters. Average daily temperatures generally ranged from 11°C– 27.5°C throughout the year and the average annual precipitation was 131 cm [29].

Ichauway consists of approximately 12,000 hectares of land in the Southeastern Coastal Plain primarily managed to maintain and restore the longleaf pine (*Pinus palustris*)-wiregrass (*Aristida beyrichiana*) ecosystem. Land use on Ichauway included 7,250 ha of longleaf pine forest, with the remaining area primarily consisting of slash (*P*. *elliottii*) and loblolly pine (*P*. *taeda*) forests, mixed pine-hardwood forests and lowland hardwood hammocks [30]. Pine forests were characterized by an open canopy, a sparse midstory, and a dense herbaceous understory. Management practices included prescribed fire on an approximate 2-year rotation; fires limited hardwood encroachment and resulted in a diverse herbaceous understory of wiregrass and other native ground cover species. Hardwood removal through mechanical means, such as roller chopping and logging, was also performed to maintain open canopies and promote herbaceous ground cover. Predator trapping occurred on Ichauway, with removal focused on opossums (*Didelphis virginiana*), and raccoons (*Procyon lotor*). However, coyotes, bobcats, and–prior to this study–gray foxes were sometimes removed primarily as bycatch (i.e., there was no focused effort on removing these species except during an experimental removal that occurred during the spring and summer of 2007 [31] to evaluate predator impacts on white-tailed deer (*Odocoileus virginianus*) recruitment). Importantly, coyotes and bobcats are known predators of gray foxes on our study area [10] and these larger predators were common on our study area [32], [33].

In contrast to Ichauway, surrounding lands were dominated by large center pivot agricultural fields with hardwood forests, pine forests, mixed pine-hardwood forests, pasture/food plots, and residential areas interspersed throughout. Agricultural fields were primarily planted with corn (*Zea mays*), peanuts (*Arachis hypogaea*) or cotton (*Gossypium* spp.) in the spring and harvested in the fall. Some agricultural fields were bisected by hedgerows typically comprised of hardwoods. Hardwood dominated stands in the surrounding areas were generally not actively managed, whereas some pine stands were managed for timber production or quail hunting.

### Trapping

We used MB-450-FOX/OS foot-hold traps (Minnesota Brand, Pennock, MN) and Victor 1.75 laminated offset foothold traps (Oneida Victor, Euclid, OH) to capture gray foxes. Trapping was continuously conducted from February 2014-August 2015 throughout Ichauway; however, periodic breaks ranging from 2 days to 3 weeks occurred due to personnel limitations. We restrained captured gray foxes using a catch-pole and secured the animal by placing electrical tape around the rostrum and legs. We used a blindfold to reduce animal stress. Weight, age (juvenile or adult), sex, reproductive condition and basic measurements (total body length, tail length, hind-foot length and ear length) were recorded. We used tooth wear, weight [34] and facial markings [35] to determine whether captured animals were adults or juveniles.

Each gray fox was given a unique ear tattoo and/or ear tags in both ears, and adult animals ≥ 3.6 kg were fit with a 180 g GPS Collar (GPS Logger W500, Advanced Telemetry Systems, Isanti, MN). We released collared individuals at the capture site. All trapping and handling procedures were approved by the University of Georgia Institutional Animal Care and Use Committee (IACUC #A2013 07-017-Y1-A0).

### Data collection and analysis

Initially, we programmed GPS collars to record a location every 3.25 hours (7–8 locations/day) until they dropped off 1 year after deployment. Beginning in January of 2015, we programmed GPS collars to record a location every 2 hours (12 locations/day) or every 1.5 hours (16 locations/day), until cessation of data collection (31 December 2015). We monitored foxes once every week with a 3-element Yagi antenna and hand-held radio telemetry receiver (Wildlife Materials, Carbondale, IL) to determine status (alive/dead) and general location. We downloaded location data when collars were retrieved following collar drop off or when the animal died. For some collars (n = 16), location data were remotely downloaded in the field using a laptop computer and handheld antenna (LairdTech, London, United Kingdom).

For analyses, we considered winter (i.e., breeding season) as 1 Jan– 31 Mar, spring (i.e., denning-early pup-rearing season) as 1 Apr– 30 Jun, summer (i.e., late pup-rearing season) as 1 Jul– 30 Sep, and fall (i.e., dispersal season) as 1 Oct– 31 Dec [34]. Individuals with ≥ 1.5 months of location data within a season were included in that season’s sample. For fine-scale temporal data (time of day; hereafter TOD) analysis, night and crepuscular (≤ 1 hour before and after legal sunrise and sunset, respectively) locations were classified as nocturnal locations, whereas remaining locations were considered diurnal locations. We combined nocturnal and crepuscular periods because gray foxes are most active during crepuscular and nocturnal periods [25], [36].

Gray fox habitat analyses were facilitated using ArcGIS 10.2 (ESRI, Redlands, CA). The initial landcover data for Ichauaway were based on 1:24,000 color infrared imagery that was subsequently field-validated during the early 1990’s. These data have been periodically updated as needed using GPS, proprietary digital images, and most recently 2015 NAIP imagery. To include areas adjacent to Ichauway in our analyses, we used 2011 National Land Cover Data [37] to delineate land use types and then verified classifications using site visits. For our analyses, we delineated 9 land use types: pine, mixed pine/hardwood, hardwood, agriculture, pasture/food plot, shrub/scrub, human use (i.e., areas associated with regular human activity such as buildings, lawns, parking areas, etc.), road, and wetland.

We analyzed habitat selection and diurnal refugia of gray foxes using the Euclidean distance approach of comparing average distances to each land use type for gray fox locations relative to expected values [38], [39], [40], [41], [42]. We created distance raster layers (30 × 30 m) for each of the 9 land use types using the Euclidean Distance Tool in ArcGIS. We calculated distance to the nearest representative of each land use type for each cell within the raster layers and from each gray fox location using the ‘extract’ function in the ‘Raster’ package with R software [43]. For each gray fox, we calculated distance ratios (mean observed/mean expected) for each of the 9 land use types. If the ratio for a given land use type was < 1, foxes were found nearer to the type than expected, and if the distance ratio was > 1, they were found at distances greater than expected [39].

We examined selection within the home range (Johnson’s [44] third order of selection) using 2 approaches. First, we accessed selection of core areas (i.e., intensively used areas) within home ranges; we refer to this as 1^st^ level analysis. We then examined habitat associated with individual animal locations within the home range and refer to this as 2^nd^ level analysis.

We used the adehabitatHR [45] package within R [43] to construct seasonal 95% (i.e., home range) and 50% (i.e., core use area) fixed kernel polygons for each individual. For fixed kernel estimation, we used a rule-based ad hoc method for selecting bandwidth by finding the smallest 0.10 increment of *h*_*ref*_ (i.e., the smoothing parameter controlling bandwidth) that resulted in a contiguous rather than disjointed fixed kernel polygon, and contained no lacuna within the home range [46]. We sequentially reduced the reference bandwidth (*h*_*ref*_) in increments of 0.1 (0.9 *h*_*ref*_, 0.8 *h*_*ref*_, 0.7 *h*_*ref*_…0.1 *h*_*ref*_) until an estimate fractured into 2 or more polygons, and selected the smallest increment of *h*_*ref*_ that resulted in a continuous polygon as the bandwidth.

For 1^st^ level analyses, we compared distances of fox locations within the core area to distances of raster cells within the home range to the nearest representative raster cell of each habitat feature. For 2^nd^ level analyses, we compared distances of gray fox locations within the home range to the distances of raster cells within the home range to the nearest representative raster cell of each habitat type.

For the 1^st^ level analysis of habitat selection, we conducted a MANOVA to assess whether selection varied as a function of sex, season, and their interaction. For the 2^nd^ level analysis of habitat selection, each gray fox location included the time of acquisition; therefore, we performed a similar MANOVA but added TOD and associated 2-way interactions between TOD, and sex and season. We used univariate t-tests to determine which habitat types were selected, not selected/avoided, or avoided. We ranked habitat types in order of preference using paired t-tests to construct ranking matrices [39].

To evaluate selection of diurnal refugia, we identified coordinates of consecutive diurnal locations that were ≤ 40 m from each other, and used the mean of these locations to generate diurnal refuge sites. For example, if 2 or more consecutive locations were separated by ≤ 40 m, the UTM coordinates were averaged and treated as a single diurnal refuge site. As above, we used MANOVA to evaluate effects of sex, season and their interaction on gray fox selection of diurnal refugia within their 95% seasonal home ranges and t-tests to determine if habitat types were selected, not selected or avoided, or avoided. We ranked habitat types in order of preference using paired paired t-tests to construct ranking matrices [39]. For all analyses of resource selection, we used the animal within a season as the sampling unit.

During diurnal VHF tracking efforts, we periodically homed in on gray foxes and flushed them from their resting site. We recorded whether collared gray foxes were diurnally resting on the ground or in a tree. We conducted homing no more than once a month on an individual gray fox to limit disturbances that may have affected habitat selection.

## Results

We collected 56,920 GPS locations from 34 (20M, 14F) gray foxes. Overall GPS fix success rate was 86.14%. At both 1^st^ and 2^nd^ levels of selection, season and sex did not interact (*F*_3,51_ ≤ 1.089, *P* ≥ 0.362) to influence habitat selection, and selection was similar (*F*_3,55_ ≤ 1.184, *P* ≥ 0.259) across seasons and between sexes (*F*_1,57_ ≤ 0.878, *P* ≥ 0.551). At the 2^nd^ level of selection, season and TOD (*F*_3,110_ = 0.831, *P* = 0.710), and sex and TOD (*F*_1,110_ = 0.805, *P =* 0.847) also did not interact to influence selection and habitat selection did not vary (*F*_1,116_ = 1.293, *P* = 0.249) by TOD. Because habitat selection did not differ with regard to sex, season, and TOD we used composite data for remaining analyses of 1^st^ and 2^nd^ levels of selection. Habitat selection was non-random (*P* < 0.001) at both levels of selection (Table 1). Gray foxes locations at both 1^st^ and 2^nd^ levels of selection were closer (*P* < 0.05) to hardwood, human use, and roads than expected; distances to other habitats were as expected (*P* > 0.05).

**Table 1:** Habitat types ranked in order of preference (1 most preferred-9 least preferred) at 2 spatial scales by gray foxes from 2014–2015 on the Joseph W. Jones Ecological Research Center at Ichauway, and surrounding lands in Baker County, Georgia, USA. Significant differences (*P* < 0.05) among ranks are indicated by different letters. S = Selected, NS = Not Selected (used as expected).

1^st^ Level Selection^a^					2^nd^ Level Selection^b^			
Habitat Type	t	P	Conclusion	Rank	T	P	Conclusion	Rank
Hardwood	-5.46	<0.001	S	1A	-4.89	<0.001	S	1A
Human Use	-3.61	<0.001	S	2A	-4.52	<0.001	S	2A
Road	-3.34	0.003	S	3A	-4.62	<0.001	S	3A
Mixed Forest	-1.87	0.074	NS	4A	-1.96	0.061	NS	4AB
Pasture/food plot	-1.01	0.324	NS	8B	-1.34	0.194	NS	6B
Agriculture	-1.67	0.108	NS	7B	-1.47	0.156	NS	7B
Pine	-2.73	0.787	NS	9B	-0.43	0.674	NS	9B
Shrub Scrub	-2.02	.0555	NS	5AB	-1.98	0.059	NS	5B
Wetland	-1.48	0.152	NS	6B	-0.99	0.330	NS	8B

^a^Selection of core areas within home range

^b^Selection of locations within home range

We grouped 4,439 diurnal gray fox locations into 59 clusters such that all points within a cluster were within 40 meters of each other. We treated these clusters as daytime refuge sites; 31 were used by males and 28 by females. Season and sex did not interact (*F*_3_,_57_ = 1.079, *P* = 0.374) to influence selection of diurnal refugia, and selection was similar across seasons (*F*_3,55_ = 1.272, *P* = 0.186) and sexes (*F*_1,57_ = 1.537, *P* = 0.166). Diurnal refugia selection was non-random (*P* < 0.001) and gray foxes were found nearer (*P* < 0.05) to hardwood, road, agriculture, human use, pastures/food plots and shrub scrub than expected; distances to other habitats were as expected (*P* > 0.05) (Table 2). During diurnal homing, we recorded 108 observations (67M, 41F) from 28 collared gray foxes (17M 11F). All gray foxes were observed on the ground.

**Table 2:** Habitat types ranked in order of preference (1 most preferred-9 least preferred) for selection of diurnal refugia by gray foxes from 2014–2015 on the Joseph W. Jones Ecological Research Center at Ichauway, and surrounding lands in Baker County, Georgia, USA. Significant differences (*P* < 0.05) among ranks are indicated by different letters. S = Selected, NS = Not Selected (used as expected).

Habitat Type	T	P	Conclusion	Rank
Hardwood	-16.56	<0.001	S	1A
Road	-10.20	<0.001	S	2A
Agriculture	-4.47	<0.001	S	3A
Human Use	-10.73	<0.001	S	4A
Pasture/food plot	-4.96	<0.001	S	5B
Shrub Scrub	-2.21	0.037	S	6B
Mixed Forest	-0.42	0.678	NS	7B
Pine	-0.44	0.665	NS	8B
Wetland	-0.07	0.948	NS	9B

## Discussion

Similar to other studies [9], [10], we found little variation in season and sex-specific habitat selection by gray foxes. Gray foxes also selected habitats similarly during diurnal and nocturnal periods, suggesting they have similar resource needs throughout the diel period or they have temporally-specific resource needs (e.g. food availability) that occur in association with selected habitat types. Further, selection of habitats associated with diurnal refugia was similar to 1^st^ and 2^nd^ level habitat selection, but more habitat features were selected for refugia, providing evidence that daytime refugia were perhaps more influenced by edge than by the specific habitat features creating the edges [40]. Interestingly, some foxes frequently used agriculture fields during the growing season for daytime refugia, but these sites were seldom used by active animals. We suggest use of monotypic agriculture fields as refuge sites allowed foxes to more easily detect and respond to potential threats.

Hardwood forests, human use, and roads ranked highly at both levels of analyses. Furthermore, although sometimes not significant, observed distances to all habitats were less than expected (i.e., all had negative t-statistics), providing evidence that edges are important to gray foxes [40]. Edges provide abundant prey and facilitate foraging [47]. Edges also may provide travel corridors [48] and although coyotes and bobcats also use these areas as travel corridors [49], [50], cover associated with edges may facilitate gray fox escape when encountering one of these larger predators.

Similar to a prior study on our study site [10], hardwoods were important to gray foxes at both levels of selection and for use as daytime refugia. Other studies suggested gray foxes are closely associated with forests [51] and where available, hardwood forests were consistently preferred [9], [10]. Chamberlain and Leopold [9] found small mammal abundance strongly affected gray fox habitat selection. Therefore, hardwood forests on our study site may have provided sufficient prey for gray foxes. Additionally, many hardwood tree species such as live oak (*Quercus virginiana*), water oak (*Quercus nigra*), and laurel oak (*Quercus laurifolia*) have large sprawling branches that may provide better arboreal escape cover from coyotes than pines. Gray foxes also forage arboreally [51], [52] and hardwood trees may provide opportunities to predate birds, bird nests, insects and squirrels.

Pine forests were used at random and were generally poorly ranked (8^th^ or 9^th^) in all ranking matrices. Wildlife management practices associated with pine forests, such as supplemental feeding of Northern Bobwhite, resulted in abundant small mammal populations which supported larger predators such as coyotes and bobcats [53]. On our study site, a lack of escape cover within pine forests may have created an environment that was too risky for gray foxes. Additionally, pine forests on our study site were burned at approximately 2 year intervals; such frequent fire limits production of soft mast [54], which is an important food for gray foxes [22] and retards hardwood encroachment. Thus, lack of soft mast and reduced hardwood within mature pine forests may explain why these forests were not selected by gray foxes [25].

Interestingly, although most gray foxes (97%) were captured on Ichauway, only 14% of all fox locations were recorded on Ichauway. In comparison, Temple et al. [10] captured all of their study animals on Ichauway and 35% of all gray fox locations occurred on the property. Chamberlain and Leopold [9] found suggested that removal of hardwoods in these forests may be detrimental to gray fox populations. Removal of mature hardwoods from within the longleaf pine matrix [55], [56] occurred since the Temple et al. [10] study and this removal may be the reason for reduced gray fox use of the property. Similarly, hardwood removal resulted in reduced use by raccoons [55] suggesting hardwood removal from within pine dominated forests may be an alternative to traditional lethal control in limiting nest predation within this forest type.

As observed by others [10], [11], [18], areas near human use were important to gray foxes. Increased human activity may have resulted in larger predators such as coyotes and bobcats avoiding these areas [11], [57], [58], [59], [60] [61]; whereas, gray foxes may be more likely to occur [62]. Additionally, human presence contributes to availability of anthropogenic food sources (e.g., garbage and pet food [18]), which may benefit gray foxes. We suggest areas near human structures provide gray foxes with habitat that both reduces predation risk and provides enhanced foraging opportunities.

All analyses suggested roads were important to gray foxes. Public roads have far more traffic than private roads on the study area, and gray fox home ranges were generally associated with public roads. These roads may be less used by bobcats and coyotes because both species avoid paved roads [11], [50] and areas with high levels of human activity [60]. Further, coyote transients frequently use areas along the boundaries of territorial coyote residents [61], [63], [64]. Thus, like transient coyotes, gray foxes may use areas near roads to avoid competition and potential predation from resident coyotes.

As with raccoons [55], [65], some [66] have suggested that gray foxes rest in trees, but limited evidence exists for such behavior [67]. We found no evidence of gray foxes diurnally resting in trees, yet they were often found in areas with low, sprawling branches that may facilitate climbing, and gray foxes may have climbed these trees to flee predators. It is also possible that foxes sought refuge in trees but abandoned their arboreal refuge when approached by a researcher. Regardless of whether gray foxes routinely seek refuge in tree canopies, we suggest hardwoods are important to the species in the southeastern United States because they provide both foraging opportunities and escape cover.

Red foxes avoid aggressive encounters with coyotes by maintaining home ranges along the boundaries or outside of coyote territories [68]; thus minimizing spatial overlap with coyotes and avoiding risky areas. In contrast, kit foxes (*Vulpes macrotis*) use underground dens to facilitate escape from aggressive encounters [69]. Our data suggest gray foxes may both avoid risky areas and utilize escape cover. Similar to kit foxes, gray foxes may use habitat elements (i.e. dense cover and trees with branch structure that facilitates climbing) within coyote territories [11], [21], and they may also use developed roads and areas near human dwellings to minimize spatial overlap with coyote territories. Interestingly, in treeless landscapes with few human dwellings, gray foxes suffer greater mortality rates from coyotes [27], [70].

## Supporting information

S1 Appendix (XLSX)
